# Synchrotron X-Ray Imaging and Spectroscopy in Soil Improvement and Remediation: A Review and Perspective

**DOI:** 10.3390/nano16080456

**Published:** 2026-04-13

**Authors:** Cheng Chen, Limin Zhou, Xingya Wang, Airong Liu, Lijuan Zhang, Jun Hu

**Affiliations:** 1 Institute of Materiobiology, College of Science, Shanghai University, Shanghai 200444, China; 23720235@shu.edu.cn; 2 Shanghai Synchrotron Radiation Facility, Shanghai Advanced Research Institute, Chinese Academy of Sciences, Shanghai 201204, China; zhoulm@sari.ac.cn (L.Z.); wangxingya@sari.ac.cn (X.W.); 3 Shanghai Institute of Applied Physics, Chinese Academy of Sciences, Shanghai 201800, China; 4 State Key Laboratory for Pollution Control and Resource Reuse, College of Environmental Science and Engineering, Tongji University, Shanghai 200092, China; liuairong@tongji.edu.cn; 5 University of Chinese Academy of Sciences, Beijing 100049, China; 6 Xiangfu Laboratory, Jiashan 314102, China

**Keywords:** synchrotron radiation, soil, heavy metal contamination, aggregates, microorganisms

## Abstract

Soil contamination by heavy metals and organic pollutants presents significant challenges to the global environment and public health. However, a lack of micro-scale understanding of the pollution process hinders efforts to remediate and enhance soil quality. Synchrotron-based X-ray imaging and spectroscopy techniques are powerful tools in revealing complex interactions within heterogeneous soil systems. This review systematically explores recent advances in soil research that deepen our knowledge on the chemical states, spatial distribution, and dynamic interactions of heavy metals and organic contaminants via synchrotron-based techniques (e.g., micro-XRF imaging, FTIR, SR-μCT). It highlights the potential of these methods to characterize composition, aggregate structure, and microbial activity within soil matrices with high spatial and temporal resolution, in situ, and with element-specific analysis. Additionally, a forward-looking perspective outlines key research directions to leverage these advantages and develop more effective and sustainable soil restoration strategies. We hope this work emphasizes the role of synchrotron science in field-scale soil applications and inspires future, mechanism-driven, evidence-based soil remediation efforts.

## 1. Introduction

Soil is a vital natural resource on Earth, playing a central role in maintaining the health of global ecosystems. As a primary interface of the land surface, it supports essential ecological functions, including agriculture, biodiversity, water cycle regulation, and carbon storage [1,2,3]. Soil health directly impacts the performance of these ecological services: it affects crop yield and quality and influences environmental processes. Soil degradation and pollution pose serious global environmental challenges, threatening ecosystem stability and agricultural productivity. The decline in soil quality—caused by industrial contamination, excessive use of agrochemicals, and unsustainable land management—undermines critical soil functions such as nutrient cycling, water filtration, and carbon sequestration [4,5,6]. For example, chemical industrial parks along the middle Yangtze River have led to significant accumulation of heavy metals (Ni, Cd, and Cu) in nearby soils, creating serious environmental and health risks [7]. The use of industrial wastewater for irrigation has further contaminated surrounding agricultural soils with heavy metals and pesticides, harming rice production and posing potential toxic risks to human health [8]. Additionally, intensive mechanized agriculture causes subsoil degradation and compaction, reducing water infiltration, nutrient efficiency, and overall soil health [9]. Therefore, soil remediation and enhancement strategies are essential for restoring ecological balance, supporting sustainable food production, and reducing environmental risks, with the combined interaction of these soil properties facilitating key ecosystem functions. However, the inherent properties of soil are often not directly measurable and must be inferred through proxy indicators to assess their related ecological processes. Consequently, a systematic evaluation of soil property indicators is crucial for improving our understanding and promoting sustainable management of soil systems.

**Figure 1:**
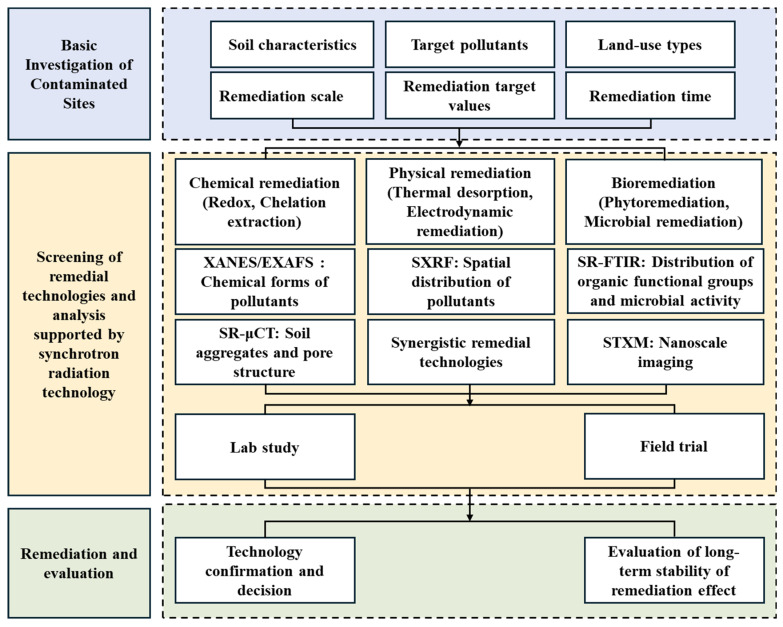
System Flowchart for Soil Pollution Site Remediation Investigation, Technology Screening, and Assessment.

Soil assessment involves the systematic analysis and evaluation of soil physical, chemical, and biological properties to determine its quality and functional suitability for agricultural, environmental, and land-use applications [4,10,11]. Given the expanding recognition of soil multifunctionality, establishing a scientifically robust and comprehensive assessment framework has become an important yet non-trivial task. As shown in Figure 1, such a framework should extend beyond conventional physico-chemical characterization to incorporate quantitative evaluations of ecological processes and ecosystem services provision, thereby supporting evidence-based strategies for sustainable agriculture and environmental conservation.

Soil health is a multifaceted concept influenced by physical structure, chemical composition, and biological activity. These properties operate across extensive spatiotemporal scales, ranging from molecular interactions within organic matter and mineral surfaces to broader landscape patterns of soil types and land use. Synchrotron-based techniques, including micro-computed tomography, X-ray absorption spectroscopy, and X-ray fluorescence microscopy, offer unparalleled spatial resolution, enabling the visualization and characterization of soil components at sub-micrometer to micrometer scales. For instance, micro-CT provides detailed insights into pore network architecture, aggregate stability, and the distribution of roots and microbes within soil aggregates—information critical for understanding water infiltration, gas exchange, and nutrient cycling. However, these microscale “hot spot” observations must be contextualized through targeted sampling and multi-scale experimental designs and integration with complementary techniques such as Raman spectroscopy to effectively link them to bulk soil health indicators.

Soil health metrics typically encompass indicators related to soil’s capacity to function as a vital living system, including nutrient cycling, water regulation, biodiversity support, and carbon sequestration [12,13]. Microscale insights into soil organic matter structure provided by synchrotron techniques can be directly linked to soil stability and long-term carbon storage potential—a key soil health indicator. Similarly, understanding microbial distribution and activity in specific microenvironments informs overall microbial diversity and functional resilience, both critical for soil health [14]. Microscale observations contribute to understanding soil “condition” and its “capacity” to deliver essential functions, while upscaling efforts are crucial for evaluating “capital” (the stock of soil resources) and “connectivity” (interactions between soil and other environmental compartments). Therefore, although synchrotron methods provide unprecedented detail at the microscale, their utility for assessing aggregate soil health and informing ecological restoration strategies is maximized when integrated into a multi-scale research paradigm that explicitly addresses spatial heterogeneity and employs robust upscaling techniques.

Synchrotron facilities worldwide serve as critical platforms for advanced research in physics, chemistry, biology, materials science, and environmental science [15,16]. Major facilities include the European Synchrotron Radiation Facility (ESRF, Grenoble, France), Advanced Photon Source (APS, Lemont, IL, USA), SPring-8 (Sayo, Japan), PETRA III (DESY, Hamburg, Germany), Diamond Light Source (Didcot, UK), Shanghai Synchrotron Radiation Facility (SSRF, Shanghai, China), and MAX IV (Lund, Sweden). These facilities provide highly brilliant, tunable, and polarized X-rays, enabling experiments impossible with conventional laboratory sources. Fourth-generation sources, such as the Extremely Brilliant Source (ESRF-EBS) and China’s High Energy Photon Source (HEPS), represent the cutting edge of this technology [17,18].

Access to beamtime is granted through a competitive peer-review proposal mechanism, free of charge for non-proprietary research. Researchers submit proposals outlining scientific merit, technical feasibility, and safety considerations, typically covering travel and sample preparation costs themselves. These facilities annually serve thousands of researchers, support numerous projects, and generate substantial scientific output. New users are encouraged to contact beamline scientists during proposal development to optimize experimental design and ensure technical feasibility.

Traditional chemistry lacks the accuracy and sensitivity required to resolve soil heterogeneity. Spectral techniques partially fill this gap: mid-infrared identifies functional-group architecture of organic matter, whereas fluorescence quantifies its concentration and reactivity. Yet laboratory-grade light sources provide narrow, incoherent spectra at λ ≥ 400 nm, affording lateral resolutions >10 µm and suffering from intensity drift that degrades long-duration mapping. Synchrotron radiation, by contrast, delivers continuous, brilliant beams down to 0.01 nm, enabling hard-X-ray nano-probes that couple elemental, structural and morphological contrasts. When combined with minimal-disturbance sample preparation—cryo-plunge fixation, ultra-microtomy, or in situ humidity cells—these probes merge molecular, particulate and core-scale information into a single, self-consistent dataset, thereby closing the observational gap between micro- and macro-scale soil processes.

**Figure 2:**
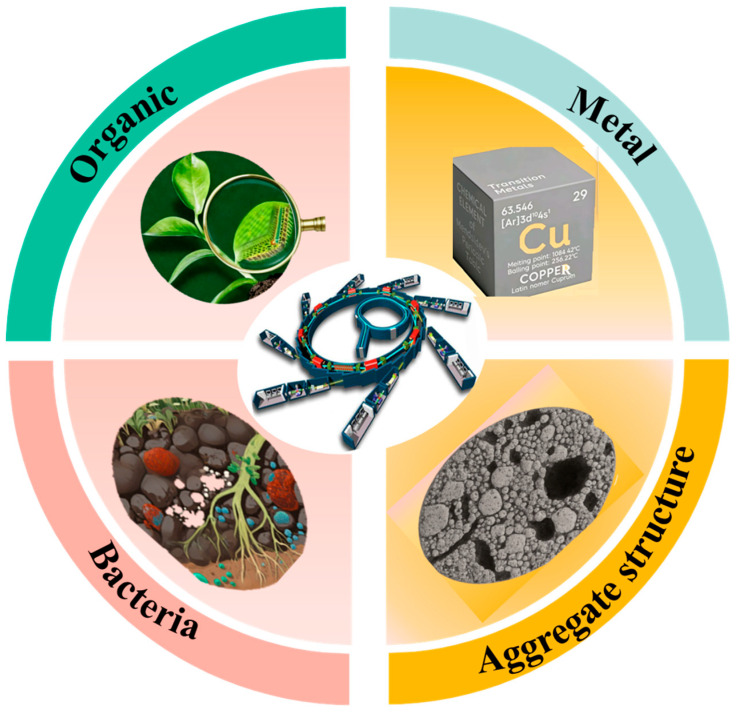
The application of synchrotron radiation in various fields of soil research.

This paper systematically reviews the latest applications of synchrotron radiation techniques in soil improvement and remediation (Figure 2 and Table 1). This review demonstrates the capability of synchrotron-based techniques—including X-ray absorption, fluorescence, and microscopy, as well as infrared spectroscopy—for elucidating the spatial distribution and chemical states of different metals in soil. The pore structure and activity of organic matter in soil revealed by synchrotron radiation micro-CT and FTIR are also discussed. Finally, we offer some perspectives on the future directions of applying synchrotron radiation techniques in multi-scale soil process research, providing a foundation for the development of intelligent and precise soil remediation systems.

## 2. Advancements in SR-Based Research Techniques

Synchrotron radiation is extremely bright electromagnetic radiation produced by relativistic electrons being deflected in a magnetic field, with brightness 10^4^–10^6^ times that of laboratory light sources. Its spectrum is continuously tunable from infrared to hard X-rays, and its spatial coherence allows it to be focused to <100 nm [29,30].

Leveraging the energy-tunability of synchrotron X-ray absorption spectroscopy, scientists can now acquire element-specific electronic and structural fingerprints in situ at ppm-level sensitivity without disturbing the sample or its native moisture, temperature, or redox state. Coupled with parallel infrared, mass-spectrometric, and microscopic readouts, these techniques are interpreted through AI-driven data mining. Synchrotron radiation thus anchors a multiscale analytical framework that links molecular-scale speciation to aggregate-scale biogeochemical function, making synchrotron radiation an indispensable diagnostic for assessing soil health and guiding sustainable land management.

### 2.1. Scanning Transmission X-Ray Microscopy

**Figure 3:**
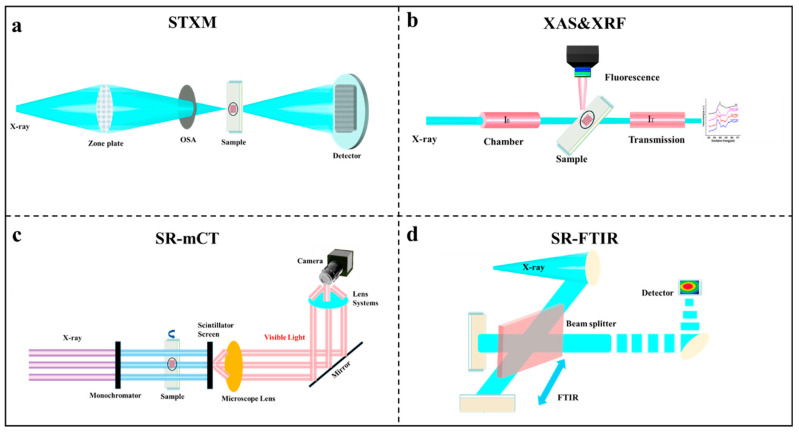
Schematic illustration of five major spectroscopic technologies. (**a**) Scanning transmission X-ray microscopy (STXM), (**b**) X-ray absorption spectroscopy (XAS) and Synchrotron-based X-ray fluorescence (SXRF), (**c**) Synchrotron Radiation-based X-ray computed tomography (SR-CT), (**d**) Synchrotron Radiation-based Fourier Transform Infrared Microspectroscopy (SR-FTIR).

Scanning Transmission X-ray Microscopy (STXM) uses synchrotron soft X-rays as a high-brightness, highly coherent light source, and focuses the X-rays to a spot smaller than 50 nm through the zone plate. The setup is shown in Figure 3a: the sample is placed at the focused spot, and by moving the sample (or the spot) in a step-scan manner, the transmitted intensity is recorded point by point. Each pixel corresponds to the absorption signal resulting from the interaction of an X-ray beam with the sample, thereby constructing a two-dimensional absorption image [31,32]. Through the six-axis collaborative upgrade of light source, optics, detection, sample environment, software and algorithms, STXM has evolved from the traditional absorption contrast scanning mode into a three-dimensional imaging operating system of spatial structure [33], time and chemical state, systematically meeting the high-precision characterization needs across multiple disciplines, becoming an essential cross-scale research platform in the soft X-ray field [34].

STXM is frequently coupled with Near-edge X-ray Absorption Fine Structure (NEXAFS) spectroscopy to enable simultaneous morphological imaging and chemical speciation analysis at the nanoscale. This correlative approach—STXM-NEXAFS—provides quantitative two-dimensional maps of various carbon species (aliphatic C, aromatic C, carboxyl C, quinones) at 50 nm resolution, making it particularly powerful for investigating soil organic matter microstructure [35], organo-mineral associations, and microbial-mineral interfaces. Similarly, X-ray Fluorescence Microscopy can be combined with micro-XANES to spatially resolve elemental distribution and chemical speciation of heavier elements at micrometer to sub-micrometer scales. These correlative approaches are essential for linking morphology and microstructure with chemical/speciation information, particularly for elements central to soil function such as C, N, and O that require soft X-ray beamlines.

### 2.2. X-Ray Absorption Spectroscopy

X-ray Absorption Spectroscopy (XAS) is a powerful analytical technique used to investigate the elemental composition, electronic states, and local atomic structure of materials. As illustrated in Figure 3b, it is based on the absorption of X-rays as they interact with matter. When X-rays pass through a sample, atoms within the sample absorb the X-rays, leading to X-ray attenuation. The degree of attenuation is closely related to the composition and structure of the sample, providing critical information about its properties. To obtain high-quality XAS data, synchrotron radiation sources are typically employed due to their high intensity and broadband X-ray energy.

XAS integrates two complementary techniques: X-ray Absorption Near-Edge Structure (XANES) and Extended X-ray Absorption Fine Structure (EXAFS). XANES focuses on the energy region near the absorption edge, where resonant absorption occurs as incident X-rays approach or slightly exceed the ionization energy of core electrons. This results in a sharp change in the absorption coefficient, providing insights into the chemical environment of the absorbing atom, such as its oxidation state, symmetry, and unoccupied electronic states. Beyond the absorption edge, EXAFS examines the higher-energy region, where oscillatory patterns in the absorption spectrum encode detailed local structural information, including the number of coordinating atoms and the degree of disorder in the coordination environment. XANES and EXAFS offer a comprehensive understanding of both the electronic and structural properties of materials at the atomic level [36].

### 2.3. X-Ray Fluorescence Spectrometry

Synchrotron-based X-ray fluorescence (SXRF) spectroscopy is an advanced elemental analysis technique based on synchrotron radiation sources. It combines the unique advantages of synchrotron radiation, such as high brightness, high collimation, and high polarization, to provide unprecedented analytical capabilities for elemental composition in various samples [37]. As a rapid and non-destructive analytical method, SXRF can perform quantitative analysis of samples in various forms—including solids, liquids, gases, and even biological tissues—under normal temperature and pressure conditions. Its detection limits can reach the ppm (parts per million) to ppb (parts per billion) range, and under optimized conditions, even lower detection limits can be achieved. The physical principle of SXRF is fundamentally similar to XAS, as both are based on the excitation and relaxation processes of inner-shell electrons in atoms. As shown in Figure 3b, when high-energy synchrotron X-rays irradiate a sample, if the incident photon energy is greater than or equal to the binding energy of an element’s inner-shell electrons, these electrons are excited to higher levels or completely ionized, creating an electron vacancy. Subsequently, outer-shell electrons rapidly transition to fill this vacancy, releasing characteristic X-ray fluorescence with specific energies. In addition, SXRF can simultaneously record multiple elements from Na (Z = 11) to U (Z = 92) in a single scan, and collect data point by point to convert it into a two-dimensional map with a resolution of up to the micrometer level, revealing the heterogeneity of microscopic elements.

### 2.4. Micro-Computed Tomography

Spatial resolution is a critical performance metric for micro-CT systems. Laboratory systems typically achieve 0.5–50 μm resolution—significantly superior to clinical CT scanners at 0.5–1 mm—enabling detailed microstructural analysis of diverse biological and material samples, including bone, calcified plaques, contrast-enhanced soft tissues, and porous materials [38]. Contrast limitations are equally important; while mineralized tissues offer excellent native contrast due to high X-ray attenuation [39], low-density samples often require contrast agents or advanced techniques such as phase-contrast and dark-field CT [40]. Synchrotron radiation-based X-ray computed tomography (SR-CT) offers significantly enhanced spatial resolution, superior signal-to-noise ratios, and faster acquisition times, making it particularly suitable for investigating fine-scale soil structures. These advantages are most pronounced when high spatial resolution (<5 μm) and high contrast sensitivity are required for small, radiation-stable samples such as soil aggregates and rock cores. As shown in Figure 3c, X-rays from a monochromator with adjustable output energy irradiate the sample with a parallel beam, and the signal is then converted into visible light, magnified by optical lenses, and recorded by an sCMOS camera. After rotating the sample and collecting thousands of projections, a reconstruction using the inverse Radon algorithm is performed to obtain a three-dimensional absorption and phase distribution with submicron resolution [41]. This powerful combination allows researchers to characterize the multi-scale heterogeneity of soil pore systems, evaluate their hydraulic and mechanical properties, and understand how soil structure influences fundamental processes such as water infiltration, solute transport, root penetration, and microbial activity in the complex soil environment [42,43,44].

### 2.5. Fourier Transform Infrared Micro-Spectroscopy

Synchrotron Radiation-based Fourier Transform Infrared Microspectroscopy (SR-FTIR) is an advanced analytical technique that leverages the high brightness of synchrotron radiation sources combined with the high sensitivity and broad dynamic range of Fourier transform infrared spectrometers [45]. As shown in Figure 3d, after being transported and focused in vacuo, the light beam irradiates the sample. By detecting transmitted, reflected, or absorbed signals, information on molecular vibrations, lattice phonons, free carrier responses, and other properties can be obtained for in situ dynamics, micro-region imaging, and studies of light-induced processes [46,47,48]. SR-FTIR can also acquire chemical spectra and spatial distribution simultaneously through mapping, enabling the investigation of microbial distribution in plant roots. With continuous technological advancements, SR-FTIR has evolved from infrared spectral imaging at the tissue level to imaging at the cellular level, expanding its applications in biomedical research [49,50,51].

## 3. Distribution and Speciation Analysis of Pollutant Metals in Soil

The accumulation of potentially toxic metals in soils—resulting from anthropogenic activities including industrial emissions, agricultural practices, and improper waste disposal—has raised significant environmental and ecological concerns. Elevated metal concentrations can disrupt soil biogeochemical cycles, impair microbial functions, and pose risks to human health through trophic transfer. Traditional analytical methods, such as sequential extraction and bulk spectroscopy, often fail to provide accurate speciation data or spatially resolved information, which limits our understanding of metal bioavailability and mobility. In contrast, synchrotron-based XAS techniques—particularly XANES and EXAFS—have become indispensable tools. These methods enable in situ characterization of metal speciation and distribution at micron to submicron scales, even in complex soil matrices. Coupled with XRF mapping, they offer valuable insights into metal behavior in soils.

### 3.1. Al

Aluminum (Al), one of the most abundant elements in the Earth’s crust and a major component of soil, constitutes approximately 8% of total soil elements [29]. Its behavior in soil is closely linked to soil acidification and mineral dissolution, which release Al into the soil solution, making it available for plant uptake. In acidic soils, free iron oxides and aluminum oxides are the dominant types of oxides, and their forms and transformations critically influence plant growth [52,53]. The low K-edge energy of Al (1560 eV) necessitates the use of soft X-ray beamlines for its detection, requiring analysis to be conducted in a vacuum environment. Synchrotron-based techniques, such as XANES and XRF, have emerged as powerful tools for studying Al distribution and speciation in soil and vegetation. For instance, Ildefonse et al. used K-edge XANES to investigate the coordination structure of Al in imogolite and allophane isotopes [54], while Doyle et al. identified gibbsite-like Al coatings on quartz and feldspar particles in loess [55]. Prietzel et al. further distinguished between organically bound Al and inorganic mineral-bound Al, noting a decrease in organically bound Al with soil depth [56]. In plant roots, Kopittke et al. used low-energy XRF to study Al accumulation in soybean plants, revealing its primary accumulation in roots and its inhibitory effect on cell wall loosening, a critical process for root growth [57]. These findings highlight the dynamic behavior of Al in soil–plant systems and underscore the importance of advanced analytical techniques in addressing aluminum-related challenges in agriculture and environmental management.

### 3.2. Fe

Iron (Fe) is one of the most abundant elements in the global crust, constituting approximately 5.1% of its mass [58]. As a major component of many soil parent materials, Fe plays a pivotal role in soil formation and significantly influences soil physico-chemical properties. In soil, Fe primarily exists in two redox states: Fe(II) and Fe(III). The redox transformations of Fe are closely linked to other soil redox reactions, serving as critical electron acceptors or donors in microbial metabolic processes [59,60]. These transformations affect the structure and function of soil microbial communities and regulate the bioavailability of nutrients and contaminants [61]. Iron-bearing minerals exert a profound influence on soil physico-chemical conditions through various reactions, including redox processes, adsorption, deprotonation, and co-precipitation with both organic and inorganic components in the soil solution and solid phase [62,63]. Fan et al. used SXRF mapping to study the distribution of arsenic (As) in paddy soils. They found that As distribution is heterogeneous and primarily controlled by soil redox conditions and Fe hydroxides [64].

**Figure 4:**
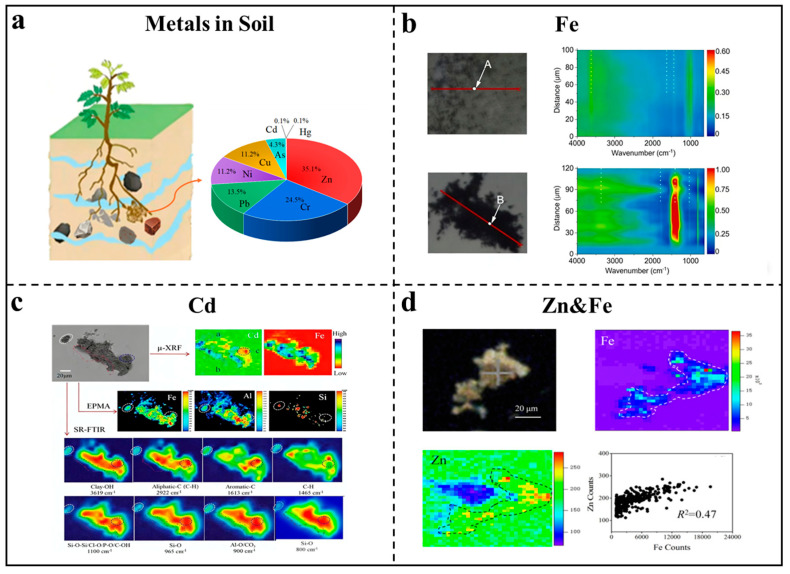
Research on Metals in Soil. (**a**) Research on Metal Elements in Soil: Distribution and proportion of major metal elements in the soil, excluding the most abundant iron and aluminum. (**b**) The micro-FTIR spectra of regions of interest (red lines in the optical photo) in the mixtures of phenanthrene with Fe-montmorillonite and goethite after 48 h incubation [68]. (**c**) Correlation analysis of multiple synchrotron radiation methods for Cd in long-term fertilized soil [69]. (**d**) SR-FTIR images of the distribution of zinc elements in soil thin sections used for fertilization [65].

Fe plays a critical role in the binding and immobilization of trace metals such as Zn. As shown in Figure 4d, Sun et al. employed FTIR and μ-XRF spectral microscopy to analyze Zn binding characteristics in soil under different fertilization conditions [65]. Their findings revealed that Zn binding sites are heterogeneous and strongly spatially correlated with submicron-scale Fe, indicating that Fe-bearing minerals provide the majority of Zn binding sites. This highlights the importance of Fe in regulating the bioavailability and mobility of trace metals in soil. The rhizosphere, the soil region influenced by plant roots, is a hotspot for iron transformations. Veelen et al. investigated rhizosphere chemistry using SXRF and XANES. They observed that Fe and sulfur (S) are primarily concentrated near plant roots, with XANES revealing that Fe is slightly reduced, S is converted to sulfate, and phosphorus (P) is gradually adsorbed onto humus. These findings underscore the complex interplay between Fe dynamics and nutrient cycling in the rhizosphere [66]. Fe also plays a crucial role in the immobilization of environmental contaminants. Xia et al. combined synchrotron-based scanning transmission microscopy, scanning diffraction coherent imaging, and nuclear magnetic resonance (NMR) techniques to elucidate the molecular mechanism by which straw organic matter promotes the co-precipitation of Fe(III) and Cr(III) for chromium (Cr) immobilization [67]. They found that soluble organic matter acts as a bridge-like structure, facilitating the co-precipitation of organic-iron oxide complexes and indirectly promoting Cr fixation. In soils co-contaminated with organic and inorganic pollutants, iron dynamics significantly influence pollutant behavior. As shown in Figure 4b, Sun et al. used chemical analysis combined with SR-FTIR and μ-XRF to analyze contaminated soil profiles [68]. They found that reactive Fe content is positively correlated with the abundance of organic-inorganic co-contaminants and soil microbial communities, thereby influencing the migration and transformation characteristics of co-contaminants.

Fe is not a contaminant in this context but rather a ubiquitous soil constituent that plays a central role in regulating organic matter dynamics. Synchrotron analyses reveal that Fe is predominantly present as oxides and sulfides, which exhibit high specific surface area and surface reactivity. This phase is particularly effective at stabilizing organic carbon through ligand exchange and co-precipitation, forming organo-mineral associations that enhance soil aggregation and carbon persistence—key indicators of soil health and critical endpoints in remediation efforts aimed at restoring functional soils.

### 3.3. Cu

Copper (Cu) is an essential trace element for plant growth and development, playing a critical role in numerous biochemical processes within cells and soil microorganisms [70,71,72,73]. Under natural conditions, Cu concentrations in soil are typically low and non-toxic. However, anthropogenic activities such as excessive mining, industrial waste disposal, and the prolonged use of Cu-based fungicides have led to widespread Cu contamination in soils. Elevated Cu levels can negatively impact soil ecosystems by inhibiting plant growth, reducing microbial activity, and impairing soil enzyme functions. Understanding the behavior, toxicity, and environmental impact of Cu in soil is essential for developing effective mitigation strategies.

Cu toxicity in plants primarily occurs through its accumulation and transport in root systems. XANES studies have provided critical insights into these processes. For example, Cao et al. used XRF to investigate the spatial distribution of Cu in willow trees exposed to adverse environmental conditions, such as flooding. Their findings revealed that Cu preferentially accumulates in the roots, and its toxicity is alleviated under waterlogged conditions [74]. This suggests that flooding inhibits Cu uptake and transport in plants, offering a potential mechanism for mitigating Cu toxicity in contaminated soils. The binding of Cu with soil organic matter (SOM) significantly influences its bioavailability and mobility in soil. Sun et al. employed FTIR to characterize the interactions between Cu and dissolved organic matter [75]. Their results demonstrated that Cu, clay minerals, sesquioxides, and carbon functional groups are heterogeneously distributed at the microscale.

The application of nanomaterials, such as carbon dots, has emerged as a potential strategy for mitigating Cu toxicity in plants. Ducic et al. utilized SR-FTIR combined with X-ray photoelectron spectroscopy (XPS) to evaluate the effects of carbon dots on Cu toxicity [76]. Their study revealed that an optimal concentration of carbon dots alleviates Cu-induced damage to polysaccharides and proteins in plant root cell walls and mesophyll tissues. Understanding the speciation and binding mechanisms of Cu in soil is crucial for predicting its behavior and toxicity. Yang et al. employed a combination of synchrotron-based techniques, including XANES, EXAFS, μ-XRF, and STXM, to investigate Cu speciation in heterogeneous soil matrices. Their results indicated that Cu primarily binds to Fe oxides, forming stable complexes [77]. This was consistent across multiple analytical techniques, demonstrating that Fe(III) oxides play a dominant role in adsorbing and immobilizing Cu in soil.

### 3.4. Cd

Cadmium (Cd) is a highly toxic heavy metal whose accumulation in soil poses significant risks to soil quality, agricultural productivity, and human health. Cd can be absorbed by plant roots, accumulate in plant tissues, and enter the food chain, increasing the risk of chronic poisoning and cancer in humans through the consumption of contaminated crops [78,79,80,81]. Cd predominantly exists as Cd(II)-O complexes in soil–plant systems, facilitating its uptake by plants and subsequent entry into the food chain. An understanding of Cd speciation, binding mechanisms, and interactions with soil constituents at the molecular scale is critical for developing effective remediation strategies.

Sun et al. employed synchrotron radiation-based micro-spectroscopy combined with two-dimensional correlation spectroscopy to investigate Cd–functional group interactions and binding sites in soil, revealing that Cd, minerals, and organic functional groups are heterogeneously distributed within soil micro-aggregates (Figure 4c) [69]. Kunene et al. used XANES/EXAFS spectroscopy to study the distribution and speciation of Cd in contaminated paddy fields and rice, and found that Cd mainly exists in the form of Cd(Ⅱ)-O in soil and rice crops [82].

### 3.5. Zn

Zinc (Zn) is an essential micronutrient for microorganisms, plants, animals, and humans, playing a critical role in biochemical processes such as photosynthesis, protein synthesis, and disease defense mechanisms. However, excessive Zn in soil can lead to phytotoxicity, causing reduced plant growth, impaired photosynthetic and respiratory activities, and increased reactive oxygen species generation [83,84,85]. To mitigate Zn pollution, it is essential to minimize Zn emissions, monitor soil Zn levels, and develop effective control strategies that enhance soil adsorption capacity and reduce Zn bioavailability. Adele et al. [86] used transmission electron microscopy and XANES to identify the forms of Zn in plant roots and found that Zn in soil mainly exists in two forms: soluble Zn species, such as zinc sulfate (ZnSO_4_), and nanoparticles, such as zinc oxide (ZnO) and zinc sulfide (ZnS). Sun et al. used synchrotron FTIR and μ-XRF to study the binding characteristics of Zn in soil and found that the distribution of Zn, clay minerals, oxides, and C functional groups at the microscale is uneven, indicating that the binding sites of Zn in soil are also uneven [65]. μ-XRF analysis showed that the distribution of Zn on soil particles has a strong spatial correlation with Fe, suggesting that iron-containing minerals provide binding sites for Zn. The chemical forms and binding sites of Zn in soil are influenced by multiple factors, including its chemical forms, functional groups in the soil, and the activity of rhizosphere bacteria, all of which collectively determine bioavailability and toxicity to plants.

### 3.6. Pb

Lead (Pb) is a highly toxic heavy metal that poses significant risks to both plant health and human safety [87]. In plants, Pb toxicity inhibits growth, disrupts physiological processes such as photosynthesis and biological nitrogen fixation, and suppresses seed germination. Moreover, Pb can enter the food chain, causing severe damage to the human nervous, digestive, and immune systems. Pb exists in various forms, including soluble Pb, Pb bound to organic matter, and Pb associated with soil minerals, with its bioavailability influenced by competitive interactions with other elements such as Mn, Fe, and Zn [83,88,89]. Li et al. used these methods to investigate Pb-tolerant bacteria in farmland soil from the Lanping lead-zinc mining area, revealing the mechanisms of Pb biosorption and transformation [89]. Similarly, Shen utilized in situ SR-μXRF combined with XANES to investigate Pb distribution and speciation, demonstrating competitive interactions with Mn. Shen et al. demonstrated that Pb competes with Mn in Arabidopsis seedlings, increasing oxidative stress and limiting Mn uptake [90]. Mera et al. employed SR-μXRF to map Pb spatial distribution in Brassica napus tissues, revealing co-distribution with Zn, P, S, and Fe. Mera et al. employed SR-μXRF to map Pb spatial distribution in Brassica napus tissues, highlighting its potential for Pb phytoremediation. Their results showed that Pb is translocated to leaves while remaining concentrated in roots, with co-distribution patterns observed between Pb and elements such as Zn, P, S, and Fe [91]. These findings underscore the importance of understanding Pb speciation, bioavailability, and plant-metal interactions to develop effective phytoremediation strategies and mitigate Pb pollution, ultimately safeguarding environmental and human health.

Collectively, these metal-specific findings demonstrate how synchrotron-derived speciation information provides a mechanistic basis for predicting contaminant behavior under field conditions, selecting appropriate remediation strategies, and designing monitoring programs that target the most labile or persistent metal pools. This approach directly supports the overarching goal of translating high-resolution molecular-scale insights into actionable guidance for soil remediation and long-term soil improvement.

## 4. Soil Pore Structure and Organic Matter Analysis

**Figure 5:**
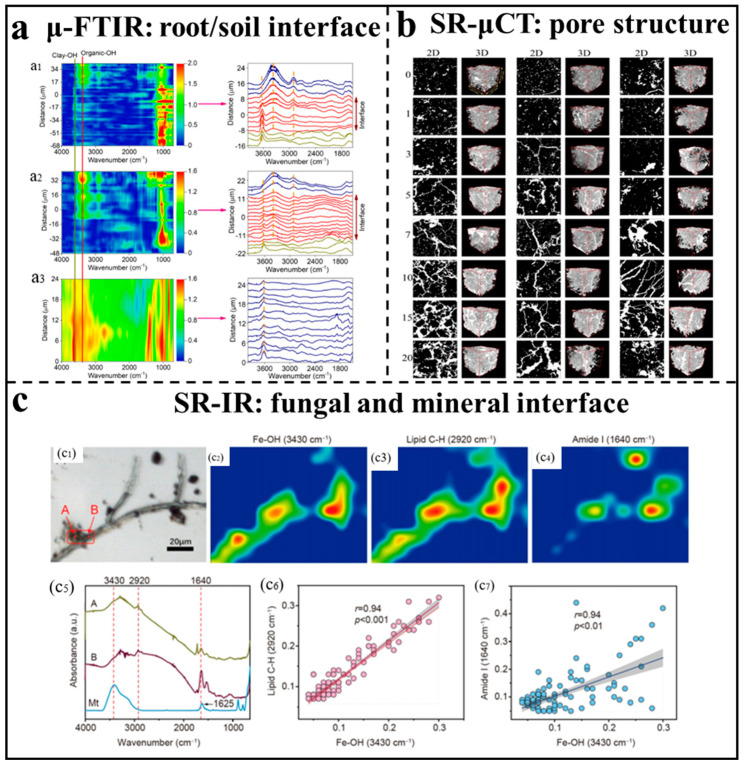
(**a**) The micro-FTIR (μ-FTIR) spectra of ROI in the bulk soil and on the root/soil interface in rhizosphere, (**a1**), (**a2**), and (**a3**) represent the rhizosphere of *Leptochloa chinensis* (L.) Nees, the rhizosphere of *Cyperus rotundus* L., and bulk soil, respectively. Step size: 2 × 2 μm² [26]. (**b**) Two-dimensional and three-dimensional visualization of soil aggregate structure under different freeze–thaw cycle counts [25]. (**c**) Characteristics of the fungal and mineral interface analyzed by SR-FTIR microscopy, (**c1**) Optical image. (**c2**–**c4**) SR-FTIR of functional groups. (**c5**) Micro-FTIR (μ-FTIR) spectra. The positions of A, B can be seen in (**c1**). Magnetite standard samples. (**c6**,**c7**) Linear correlation between functional groups [104].

Soil structure, especially the characteristics and stability of aggregates, is a key factor determining soil quality and agricultural productivity [92,93]. As the basic units of soil structure, soil aggregate pore characteristics and structural stability directly affect soil erosion resistance and water retention capacity [94,95,96,97,98]. Traditional research methods, such as electron microscopy and digital image analysis of soil thin sections, can provide two-dimensional structural information but are time-consuming and struggle to fully characterize three-dimensional spatial features [99,100,101]. In recent years, advances in computer tomography technology, particularly the application of high-resolution synchrotron radiation CT, have achieved breakthroughs in nondestructive three-dimensional imaging at the aggregate scale [25,102]. Ma et al. [25] used synchrotron radiation CT and ImageJ software to reconstruct three-dimensional microscopic images (Figure 5b) to study the effect of pore characteristics of black soil aggregates on their stability under freeze–thaw cycles. They found that total porosity, equivalent porosity, and porosity larger than 100 μm are the main factors regulating air content, water infiltration, and cohesive failure stress, and these factors collectively reduce aggregate stability. Zhao et al. used synchrotron-based X-ray micro-computed tomography to investigate the microstructural evolution of soil aggregates during natural vegetation restoration [103]. They observed a shift from small to large pores and a rapid improvement in soil microstructure, characterized by increased connectivity and porosity as vegetation recovered.

Soil structure provides physical protection and space for soil organic matter, while soil organic matter, as a binding substance, is a key factor in forming and stabilizing good soil structure. As shown in Figure 5a, Du et al. explored the chemical composition and functional group changes in organic carbon at the root-soil interface in the rhizosphere using a method combining SR-FTIR and two-dimensional correlation spectroscopy, providing a new approach for studying the structural relationship between plant roots and the soil interface [26]. They found that aggregate stability is closely related to pore characteristics. Moreover, Lu et al. explored the impact of organic and inorganic fertilization on the pore structure of paddy soil aggregates, finding that long-term application of bio-organic fertilizers enhanced porosity, pore connectivity, and complexity, whereas chemical fertilizers reduced pore system connectivity [105]. These findings underscore the critical influence of soil physical structure on the storage and utilization of organic matter. Kaestner et al. further demonstrated, using STXM, that small organic matter particles are physically trapped within micropores [106]. These insights highlight the importance of understanding soil pore structure and its interactions with organic matter for optimizing soil management practices and improving agricultural sustainability.

Combining synchrotron data with observations from other techniques that cover broader spatial scales is essential for a comprehensive understanding. For instance, initial broad-scale characterization of a soil core can be performed using laboratory X-ray CT to identify larger features. This can then be followed by targeted, high-resolution synchrotron analyses on specific sub-regions of interest at the microscale [107,108]. These integrated multi-scale observations are crucial for informing and validating multi-scale transport and reaction models that explicitly account for the heterogeneity inherent in soil systems. Developing robust statistical relationships between microscale properties and macroscale soil properties represents another approach to facilitate upscaling [109]. Such empirical relationships, derived from a diverse range of samples, can be used to estimate larger-scale behavior from microscale measurements, thereby providing a bridge from detailed observations to broader predictions.

## 5. Soil Microbial Remediation

Soil microorganisms, as indispensable components of global ecosystems, play critical roles in biogeochemical cycling, energy flow, and biogeochemical processes [110,111,112,113]. Synchrotron radiation technology, with its unique analytical capabilities, has revolutionized the in situ analysis and functional exploration of soil microorganisms by providing insights into their interactions with environmental factors. STXM-NEXAFS allows for the visualization and chemical characterization of organic matter components within soil aggregates and pores. This microstructural arrangement is critical as it dictates the physical space available for microbial colonization and activity. High-resolution imaging can delineate the spatial distribution, size, connectivity, and tortuosity of pore networks, which directly influence water flow, gas exchange, and nutrient transport—all vital for microbial survival and activity This enables the identification of “hot spots” where optimal conditions for microbial growth may exist, such as interfaces between mineral particles and organic matter, or within water-filled micropores [14].

Soil texture porosity influences microbial access to nutrients through pore networks, driving essential chemical reactions. SR-FTIR revealed that magnetite particles are closely attached to fungal hyphae and are unevenly distributed on the surface of the hyphae. It revealed that the extracellular polymeric substance matrix of the fungus exhibited a highly heterogeneous spatial distribution. This may allow the fungus to effectively decompose organic matter while protecting fungal cells and their extracellular enzymes from damage in Figure 5c [104]. Advances in microbial diversity research have revealed that soil properties such as pH, organic carbon, and inorganic nutrient concentrations are key drivers of microbial community structure and activity [114].

Furthermore, soil depth and the presence of organic-inorganic pollutants jointly shape microbial community dynamics, which in turn directly influence the migration and transformation characteristics of co-existing contaminants. Kelly et al. demonstrated that microorganisms interact with heavy metal ions, with uranium (VI) preferentially binding to phosphate groups and cadmium (II) to carboxyl groups at lower pH levels [115]. Many soil microorganisms can immobilize toxic chemicals and transform them into less toxic forms, highlighting their pivotal role in environmental remediation. However, the underlying mechanisms remain complex. Liu et al. utilized SR-FTIR to reveal the heterogeneous distribution on white-rot fungal hyphae surfaces, showing that these fungi activate oxygen vacancy formation on mineral surfaces through biomineralization, thereby driving nanozyme activity to degrade organic pollutants [104]. These findings underscore the intricate relationships between soil microorganisms, environmental factors, and pollutants, emphasizing the importance of advanced synchrotron-based techniques in elucidating these interactions and advancing microbial remediation strategies.

## 6. Conclusions and Perspective

Synchrotron radiation science stands at a critical juncture of an unprecedented technological revolution. Advancements in next-generation diffraction-limited storage ring light sources and free-electron lasers have once again enhanced the brightness, coherence, and stability of light sources. In the field of soil pollution research, synchrotron radiation technology has shifted from traditional static characterization to dynamic process and in situ analysis, achieving a cross-scale cognitive leap from molecular coordination environments to microscale interfacial reactions. Metal speciation analysis based on XAS, organic molecular structure analysis using FTIR, and elemental distribution imaging with XRF constitute a “trinity” technology system for soil pollutant research.

Currently, in situ SR techniques face numerous bottlenecks such as loss of moisture in environmental samples under vacuum conditions, beam drift effects in micro-area analysis, and self-absorption and scattering interference caused by complex matrices. These technical challenges severely limit data quality and quantitative accuracy. The challenges in data analysis are more pronounced. Real-time processing of high-dimensional hyperspectral imaging data requires novel machine learning algorithms to overcome the limitations of traditional linear fitting methods in complex multi-component systems. Deep learning networks show great potential in spectral denoising, feature extraction, and component quantification, but the lack of training data and insufficient model interpretability remain key obstacles. Furthermore, the fusion of multimodal data obtained from different synchrotron radiation techniques requires the establishment of standardized data formats and metadata specifications to achieve data comparability across platforms and scales.

In the study of environmental remediation mechanisms, nanobubble technology shows revolutionary application prospects. However, characterizing nanobubbles in soil pores is still challenging: traditional optical microscopy is limited by the diffraction limit and cannot resolve bubbles smaller than 200 nm, while electron microscopes cannot maintain the original state of water-containing samples. Synchrotron phase-contrast imaging techniques provide a breakthrough solution to this problem. By detecting the phase variation of X-rays passing through the sample, high-sensitivity imaging of nanobubble–water interfaces with very small density differences can be achieved [116]. Recent studies indicate that surface-functionalized nanobubbles can significantly enhance the desorption efficiency of heavy metals in soil, and their mechanism involves the generation of free radicals at the bubble interface and changes in the molecular structure of pollutants [117,118,119]. These microscopic processes require tracking in real time using time-resolved X-ray spectroscopy techniques.

Synchrotron radiation based X-ray technologies will drive environmental soil science toward a new era of cross-scale intelligence: by using a nanoscale in situ platform to capture real-time reactions at pollutant three-phase interfaces, integrating AI with multimodal data to achieve a full-chain closed loop of mechanism analysis, risk prediction, and remediation decision-making, and employing high-energy X-ray induced oxidative remediation technology to accomplish an integrated ‘diagnosis-treatment’ process, ultimately establishing a predictive, early-warning, and controllable smart soil health management system.

## Figures and Tables

**Table 1:** Synchrotron radiation-based analytical techniques commonly used in soil research and examples of their applications.

Technical Features & Information Obtained	Representative Applications in Soil Improvement/Remediation	Key References
X-ray Absorption Near-Edge Structure (XANES)	Elemental speciation, oxidation state, coordination environment (µm-scale resolution)	Villalba-Ayala et al. (2025) [19] Qin et al. (2025) [20] Le Pape et al. (2020) [21]
Extended X-ray Absorption Fine Structure (EXAFS)	Atomic coordination number, bond length, local structure (Å-level accuracy)	Gu et al. (2022) [22]
Scanning Transmission X-ray Microscopy (STXM)	Offer nanoscale spatial resolution combined with chemical speciation capabilities.	Xia et al. (2020) [23]
Synchrotron Radiation Micro-Computed Tomography (SR-μCT)	3-D, non-destructive, sub-micron pore structure imaging	Fatima et al. (2024) [24] Ma et al. (2021) [25]
Synchrotron Radiation Fourier Transform Infrared Spectroscopy (SR-FTIR)	High throughput, high signal-to-noise ratio; quantification of functional groups with 2-D mapping	Du et al. (2021) [26]
Synchrotron X-ray Fluorescence (SXRF, micro-XRF)	µm-scale elemental distribution maps; simultaneous multi-metal detection	Zhao et al. (2023) [27] Marafatto et al. (2021) [28]

## Data Availability

The data presented in this study are available on request from the corresponding authors.
